# Cellulose-Based Carbon Fibers: Enhanced Orientability by Boric Acid During Carbonization

**DOI:** 10.3390/polym18151894

**Published:** 2026-08-01

**Authors:** Tobias Hückstaedt, Jens Erdmann, André Lehmann, Robert Protz, Johannes Ganster

**Affiliations:** 1 Department Polymer Engineering, Fraunhofer Institute for Applied Polymer Research IAP, Geiselbergstraße 69, 14476 Potsdam, Germany; 2 Department Fiber Technologies, Fraunhofer Institute for Applied Polymer Research IAP, Geiselbergstraße 69, 14476 Potsdam, Germany

**Keywords:** carbon fibers, cellulose, boric acid, orientability, stress graphitization, catalytic effect, Rayon, structure–property relation, sustainability

## Abstract

In the present paper, a scalable and continuous process with maximum treatment temperatures of 2000 °C for making cellulose-based carbon fibers (CFs) having Young’s moduli of up to 230 GPa is presented. This unexpected high modulus was realized by using a boric acid (BA)-doped viscose precursor yarn. Such a precursor shows significantly improved orientability during carbonization, resulting in highly oriented CFs. For clarifying the underlying effect, a BA-doped and an undoped precursor (reference) were carbonized at different stretch levels, and the resulting CFs were systematically analyzed in terms of structural parameters characterizing the crystalline phase, i.e., crystallite dimensions ($L_{a}$, $L_{c}$), lattice plane spacing ($d_{002}$), and crystallite orientation ($\langle \cos^{2}\phi \rangle$). Moreover, electrical resistivity and mechanical properties were determined. It was found that BA promotes the formation of graphite-like structures and their alignment with the fiber axis. Finally, both effects result in significantly improved CF properties, particularly electrical conductivity and Young’s modulus, which are nearly three times and two times higher, respectively, than those of the reference. To explain the effectiveness of BA during thermal conversion, a microstructural mechanism is proposed based on results from a uniform stress model.

## 1. Introduction

Carbon fibers (CFs) have high strength (up to 7 GPa) and stiffness (up to 935 GPa) at a low density of usually less than 2.0 g/${cm}^{3}$ [1,2]. This combination makes them particularly interesting for applications in fiber-reinforced composites used in the mobility sector, aerospace, wind turbine blades, sports goods, and medical equipment [1,3]. These fibers are mainly produced from polyacrylonitrile (PAN)-based precursor systems, which dominate the market with a share between 96 and 98% [4].

In recent years, the focus has shifted to precursor systems based on renewable resources, e.g., bio-based PAN, lignin, and cellulose, as they have the potential to significantly reduce the carbon footprint of the resulting CFs [4,5,6,7,8]. Within this group, cellulose-based CFs are the most investigated and the most advanced, but they still have two main drawbacks compared to established PAN-based CFs [1,9,10,11,12]. The first one, the low yield, can be partially overcome by using slow heating rates [11,13], reactive atmospheres, carbon-rich fillers [14,15,16,17,18,19], and various additives [4,11,20,21,22,23,24], as shown in detail for boric acid (BA) in our previous publication [25]. The second drawback is inferior mechanical properties. The tensile strengths range typically between 0.5 and 1.5 GPa [6,26] but can be increased up to 2.8 GPa when additives like ammonium tosylate in combination with a reduced-pressure stabilization are used [4,27]. The Young’s modulus of non-graphitized cellulose-based CFs is less than 120 GPa, which is still far from PAN-based standard modulus CFs like the Toray T300 type with 230 GPa [1,11,27].

This lack in modulus of cellulose-based CFs can be overcome by increasing the preferred orientation of the crystalline phase along the fiber axis, as was demonstrated by Union Carbide with the “Thornel” series [1,28,29]. The fibers were stretched at temperatures between 2500 and 3000 °C to increase the preferred orientation, which considerably improves the Young’s modulus. In doing so, CFs with Young’s moduli up to 690 GPa and tensile strength of 3.95 GPa were obtained. A disadvantage of the so-called stress graphitization is the need for additional ultra-high temperature equipment, which is expensive, energy-consuming, and maintenance-intensive. Therefore, a central aim of this work is to enhance the overall sustainability by developing a more energy-efficient thermal treatment route while retaining the use of a biogenic precursor.

From a structural point of view, the effectiveness of stretching on the orientation at a certain tension level is affected by the density of cross-links in the typical CF morphological structure, the so-called turbostratic structure. These crosslinks have a lower thermal stability compared to the sp^2^-hybridized C−C bonds of the graphite-like planes [30] as they are present in the form of sp^3^-hybridized C−C bonds [31] and can therefore be reduced by applying high temperatures. Consequently, the orientability of CFs increases with the applied temperature, especially from 2500 °C onward, as shown by Zhang et al. for cellulose-based CFs [32].

In our previous publication, we found that BA doping increases the formation of graphite-like structures at temperatures above 1000 °C [25]. This results in considerably larger crystallites, a lower lattice plane spacing, and increased preferred orientation of the graphite-like phase along the fiber axis. All these findings point to a reduced density of crosslinks compared to an undoped system. Therefore, the BA-doped system seems well suited for an effective stretch carbonization even at temperatures lower than 2000 °C.

## 2. Materials and Methods

### 2.1. Raw Materials

For the preparation of the viscose spinning solution a softwood pre-hydrolysis kraft dissolving wood pulp (Georgia-Pacific, Atlanta, GA, USA), with a degree of polymerization of DP_Cuoxam_ = 611 and an α-cellulose content of 96.5% measured according to the TAPPI methods T206 os-63 [33] and T203 os-61 [34], respectively and carbon disulfide with 99.9% purity (Merck, Rahway, NJ, USA) was used. The spinning bath was prepared from sulfuric acid (min. 95% purity, Th. Geyer GmbH & Co. KG, Renningen, Germany), sodium sulfate (min. 98.5% purity, Carl Roth GmbH & Co. KG, Karlsruhe, Germany), zinc sulfate (min. 98% purity, VWR International GmbH, Darmstadt, Germany), and demineralized water. The spun viscose fibers were treated in an additive solution produced from orthoboric acid with a purity of 99.8% (VWR Chemicals, Radnor, PA, USA) and demineralized water. The inert gas atmosphere during the thermal conversion was generated using nitrogen and argon from Linde GmbH (Pullach, Germany), with purities of 99.8% and 99.999%, respectively.

### 2.2. Spinning of the Multifilament Yarn

The two cellulose precursors were produced according to the viscose process. The preparation of the spinning solution, the wet-spinning process, and the impregnation with BA were carried out as described in [25]. At the end, two bobbins with several hundred meters of sample were produced. One with the untreated and the other with the BA impregnated cellulose yarn (Table 1).

### 2.3. Stabilization and Low-Temperature Carbonization

Both viscose precursors were thermo-mechanically stabilized and low-temperature (LT) carbonized in two separate furnaces one after the other. The stabilization was carried out in air at temperatures below 300 °C and the LT carbonization in nitrogen atmosphere at temperatures up to 1000 °C. Relevant process parameters such as retention times, temperature profiles, and the applied tension were kept constant for the undoped reference and the BA-doped precursor and are described in more detail in our previous work [25].

### 2.4. High-Temperature Carbonization

**Figure 1:**
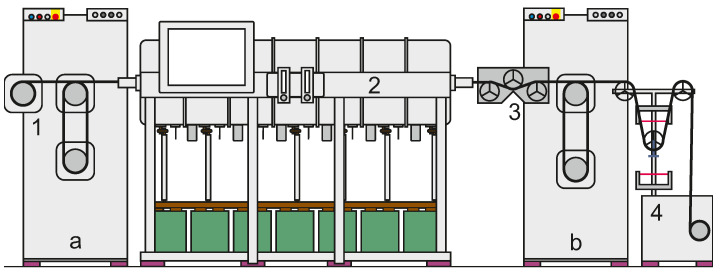
Experimental setup used for the high-temperature (HT) carbonization. Along the process chain: unwinder (1); high-temperature furnace with 6 zones (2); tension meter (3); winding head (4); rollers for fiber transport (a,b).

The high-temperature (HT) carbonization of the LT carbonized fibers based on the untreated and BA-impregnated precursors (Section 2.3) was performed on a separate processing line in argon atmosphere (Figure 1). The line speed of 0.165 m/min resulted in a retention time of approx. 3 min per zone of the furnace. The fiber passed (in Figure 1 from left to right) the six zones, which were adjusted as follows: $T_{1}=400$ °C, $T_{2}=1000$ °C, $T_{3}=1500$ °C, $T_{4}=2000$ °C, $T_{5}=2000$ °C, $T_{6}=1500$ °C. The process was started tension-free, and the stretch was incrementally increased by adjusting the speed of roller b. Samples were prepared at six different stretch levels, namely tension-free, 4.0%, 5.5%, 7.5%, 10.0%, and 12.5%. For each stretch ratio, the fiber material was completely processed through the furnace before a CF sample was taken. The resulting fiber force was recorded by a permanently installed tension meter.

### 2.5. Characterization of the CFs

#### 2.5.1. Characterization of the Cross-Sectional Area

Images of the CF single-filament fiber cross-sections were recorded by using a scanning electron microscope (GeminiSEM 300, Zeiss, Oberkochen, Germany ) at an accelerating voltage of 5 kV and a magnification of 2000×. The cross-sectional area of at least 20 different fibers was measured with the software ImageJ, version 1.51h [35]. The average cross-sectional area *A* and the corresponding errors were used to calculate the Young’s modulus, tensile strength, and the electrical resistivity.

#### 2.5.2. SEM–EDX Characterization of Fiber Surfaces

For surface morphology and defect (pores, agglomerates) analysis, CF bundles were mounted flat on sample holders with conductive carbon adhesive and sputter-coated with 4 nm of platinum (Q150V S Plus, Quorum Technologies, Laughton, East Sussex, UK). SEM was coupled with inline energy-dispersive X-ray spectroscopy (EDX) at 10 kV to determine the localized chemical composition of surface features. EDX spectra were acquired using an X-Max 80 detector and processed with AZtec software version 6.1 (both Oxford Instruments, Abingdon, UK).

#### 2.5.3. Structural Properties (WAXS)

The wide-angle X-ray scattering (WAXS) measurements were performed on a laboratory X-ray diffractometer (D8 Advance, Bruker, Billerica, MA, USA) equipped with a Cu-tube (λ=0.15419 nm) and a Si-strip detector (LYNXEYE XE-T, lynxeye, Stockholm, Sweden). The standard NIST SRM 1976c [36] was used in the Bragg–Brentano geometry for calibration. The determination of the crystallite sizes and the lattice plane spacing was carried out on milled fibers. Using a focusing Goebel mirror, θ-2θ scans were performed from 2θ=5 to 90° in transmission geometry. All diffractograms were corrected for background scattering, absorption, and polarization [37,38,39,40]. For the evaluation of the (002) [41] and the (11) [41] reflections a split Pseudo-Voigt profile was used [42]. The lattice plane spacing $d_{002}$ was calculated from the Bragg angle θ of the (002) reflection applying Bragg’s law (Equation (1)). Based on the Scherrer equation (Equation (2)), the crystallite size $L_{c}$ (graphite bundle thickness) and $L_{a}$ (persistence length parallel to the fiber axis) were evaluated using the full width at half maximum β of the (002) with K=0.89 and the (11) reflection with K=1.84, respectively [41,42,43].

(1) $$d_{002}=\frac{\lambda }{2\sin\theta }$$

(2) $$L=\frac{K\;\lambda }{\beta \cos\theta }$$

The orientation of the crystalline phase was determined from azimuthal (ϕ) scans of the (002) reflection of unidirectionally prepared fiber strands using a parallel beam with spot focus. The diffractometer was therefore equipped with a polycapillary X-ray optic combined with a collimator ( 2 mm opening). After correcting the intensities for background scattering [37,38], the ϕ-scans were fitted by applying two independent Gaussian functions on a constant background. The average of the full width at half maximum Γ of the two Gaussian functions was used to calculate the orientation degree OG according to Equation (3) and the second moment of the distribution function ($\langle \cos^{2}\phi \rangle$ according to Equation (4) [44,45]:

(3) $$OG=\frac{180{}^{\circ}-\Gamma }{180{}^{\circ}}$$

(4) $$\langle \cos^{2}\phi \rangle \approx 0.722\sin^{2}\left(\frac{\Gamma }{2}\right)$$

#### 2.5.4. Electrical Resistivity

The volume resistivity $S_{f}$ was determined on single filaments. For contacting metal clamps at four equidistant positions with a distance $L_{f}$ of 5 cm were used. The resistance between two neighboring contacts Rf was measured with a DC milliohmeter GOM-805 (Good Will Instrument Europe B.V., Veldhoven, the Netherlands) by applying a current of 10 mA. The measurement was performed 10 times per sample and $S_{f}$ was calculated from the mean of $R_{f}$ related to the fiber cross-sectional area *A* according to Equation (5):

(5) $$S_{f}=\frac{A\;R_{f}}{L_{f}}10^{3}$$

#### 2.5.5. Mechanical Properties

The tensile testing was performed on single filaments using a ZwickRoell Z020 Retro Line (ZwickRoell, Ulm, Germany) system and based on ASTM D3822 [46] in standard atmosphere (23 °C and 50% RH) according to DIN EN ISO 291 [47]. After conditioning the samples for 24 h in the testing lab, at least 15 single filaments were separated from the CF strand and tested. The following testing parameters were used: preforce of 0.1 cN, clamp distance of 20 mm and testing speed of 10 mm/min. The recorded force–strain curves were related to the average cross-sectional area *A* (from SEM) to obtain values for Young’s modulus in GPa and tensile strength in MPa.

## 3. Results

**Figure 2:**
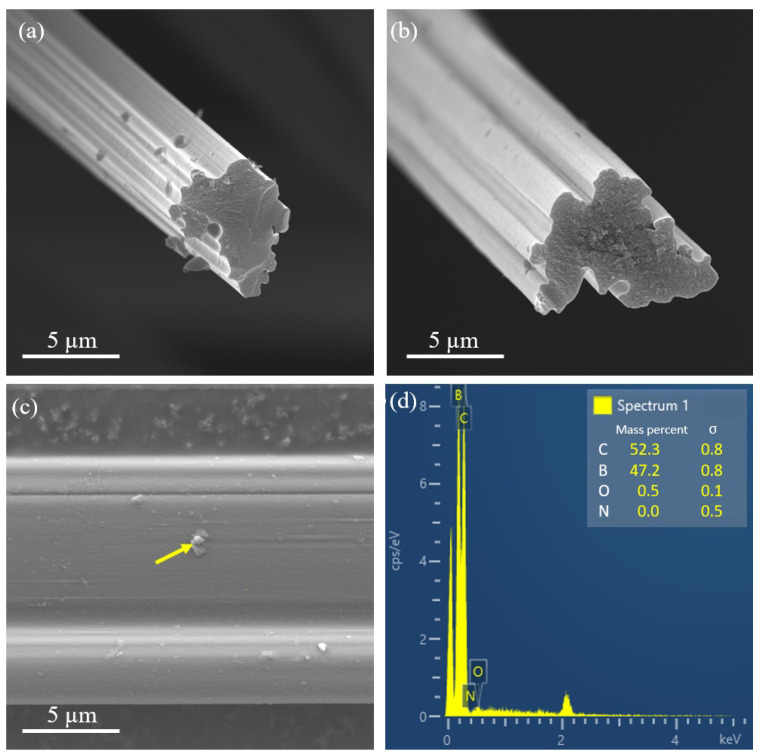
SEM images of CF of the reference system (**a**), and the BA-doped system (**b**), along with an EDX point spectrum (**d**) acquired from an agglomerate on the surface of a BA-doped CF (**c**).

As described in the Materials and Methods section, the two cellulose-based precursors, one impregnated with BA and one without (Table 1) passed successively through three thermal treatment steps, namely stabilization up to 300 °C, LT carbonization up to 1000 °C, and finally the HT carbonization up to 2000 °C. Representative SEM images of the produced CFs are presented in Figure 2.

The characteristic lobulated cross-sectional morphology of the precursors is preserved in the CF for both systems. The equivalent diameter, derived from the cross-sectional area, measures approximately 5 μm for CF of the reference system and approximately 6 μm for CF of the BA-doped system, reflecting the higher carbon yield achieved by BA impregnation [25].

### 3.1. Stretch-Tension Behavior During HT Carbonization

**Figure 3:**
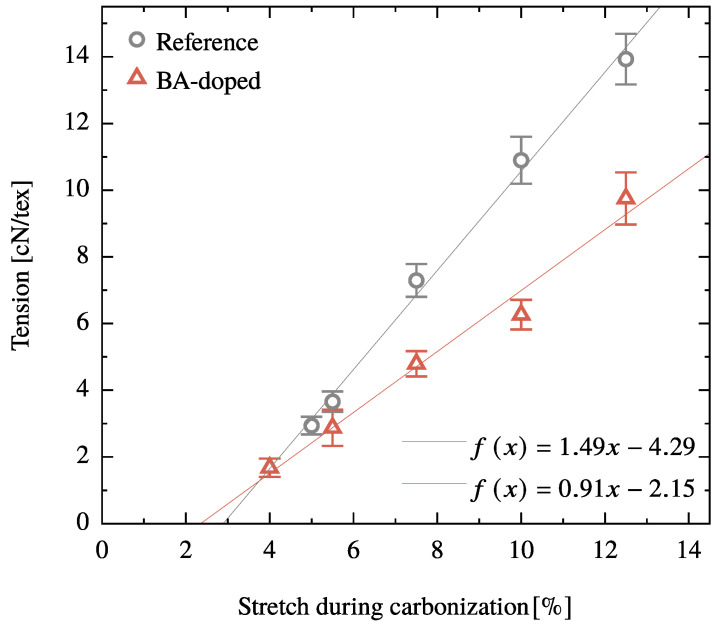
Resulting tension during HT carbonization at 2000 °C of the undoped (Reference) and the BA-doped CFs.

For the continuous HT carbonization experiments, the LT carbonized fibers (see Section 2.3) based on the untreated reference and BA-doped precursor were used. Figure 3 shows the correlation between applied stretch and the resulting fiber tension. The latter was calculated from the fiber force related to the linear density of the CFs after HT carbonization.

For both systems, a linear relation between the applied stretch and the resulting fiber tension was found. At any stretch level, the BA-doped system shows a lower tension, which is represented by an approximately 60% lower slope of the linear regression shown in Figure 3. This means that the system reacts in a more compliant way to the applied deformation. Consequently, also the maximum fiber tension at 12.5% stretch is lowered from 13.9 to 9.8 cN/tex for the BA-doped system. By extrapolating the linear regression, a positive intercept with the x-axis of 2.9% and 2.4% for the undoped reference and the BA-doped precursor, respectively, was found, indicating an intrinsic self-elongation during the HT carbonization for both systems. This unexpected behavior can be explained by the formation and orientation of crystalline structures [25], which overcompensates for an expected shrinkage of the fibers caused by mass loss and increasing density. In the following, the stretch values of 2.9% and 2.4% were used in the figures to represent the samples processed tension-free.

### 3.2. Microstructural Changes After Stretch Carbonization

#### 3.2.1. Crystalline Dimensions

**Figure 4:**
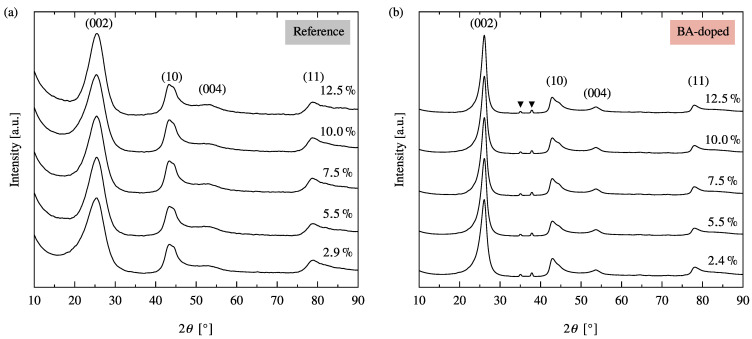
Corrected WAXS diffractograms of the CFs made from the cellulose precursor (Reference) (**a**) and the BA-doped precursor (**b**) processed at different stretch ratios during carbonization at 2000 °C.

The crystalline phase was examined using wide-angle X-ray scattering (WAXS). The corrected diffractograms show the (002), (10), (004), and (11) reflections of a graphite-like phase [45,48] (Figure 4). The shape of the reflections differs significantly for the CFs based on the untreated and the BA-doped precursor. A more pronounced formation of the crystalline phase during the HT carbonization for the BA-doped system can be derived from a shift of the (002) reflections to higher angles and a reduction of the peak width in general. In contrast to the reference system, additional reflections at 35.0° and 37.8° (marked with ▼ in Figure 4b) are found for the BA-doped system. These are attributed to a boron carbide phase formed between 1200 and 1400 °C [25,49,50].

In contrast to the remarkable changes caused by BA doping (described in detail in [25]), the impact of the stretch variation during carbonization on the crystallite sizes and the lattice plane spacing is rather small (Table 2). An increase in $L_{a}$, $L_{c}$, and $d_{002}$ was observed for both systems when a tension was applied.

#### 3.2.2. Crystalline Orientation

**Figure 5:**
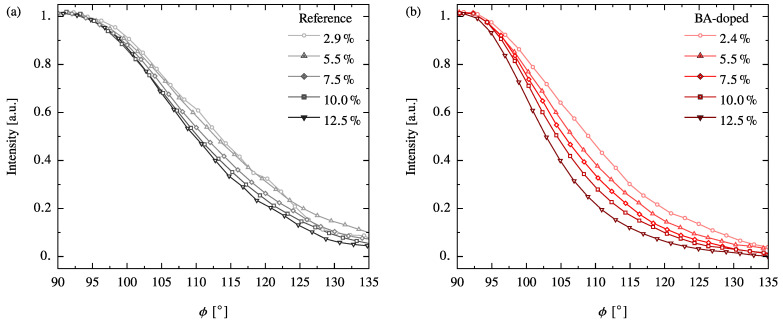
Normalized azimuthal scans of the CFs made from the cellulose precursor (Reference) (**a**) and the BA-doped precursor (**b**) processed at different stretch ratios during HT carbonization at 2000 °C.

The orientation of the crystalline phase was determined from azimuthal scans, which were normalized to the maximum intensity for better comparison (Figure 5). Three findings are derived from these scans: (i) for both systems, the orientation of the crystalline phase along the fiber axis increases with the level of the applied stretch during the HT carbonization; (ii) the increase in orientation is considerably higher for the BA-doped system; (iii) the BA-doped CFs show a smaller orientation distribution for all applied stretch ratios.

These observations were confirmed by the orientation parameters OG and $\langle \cos^{2}\phi \rangle$, which were calculated from the full width at half maximum (Γ) of the orientation distributions (Table 3).

The structural reason for the increased orientability will be discussed in Section 3.4.

### 3.3. Macroscopic Properties After Stretch Carbonization

**Figure 6:**
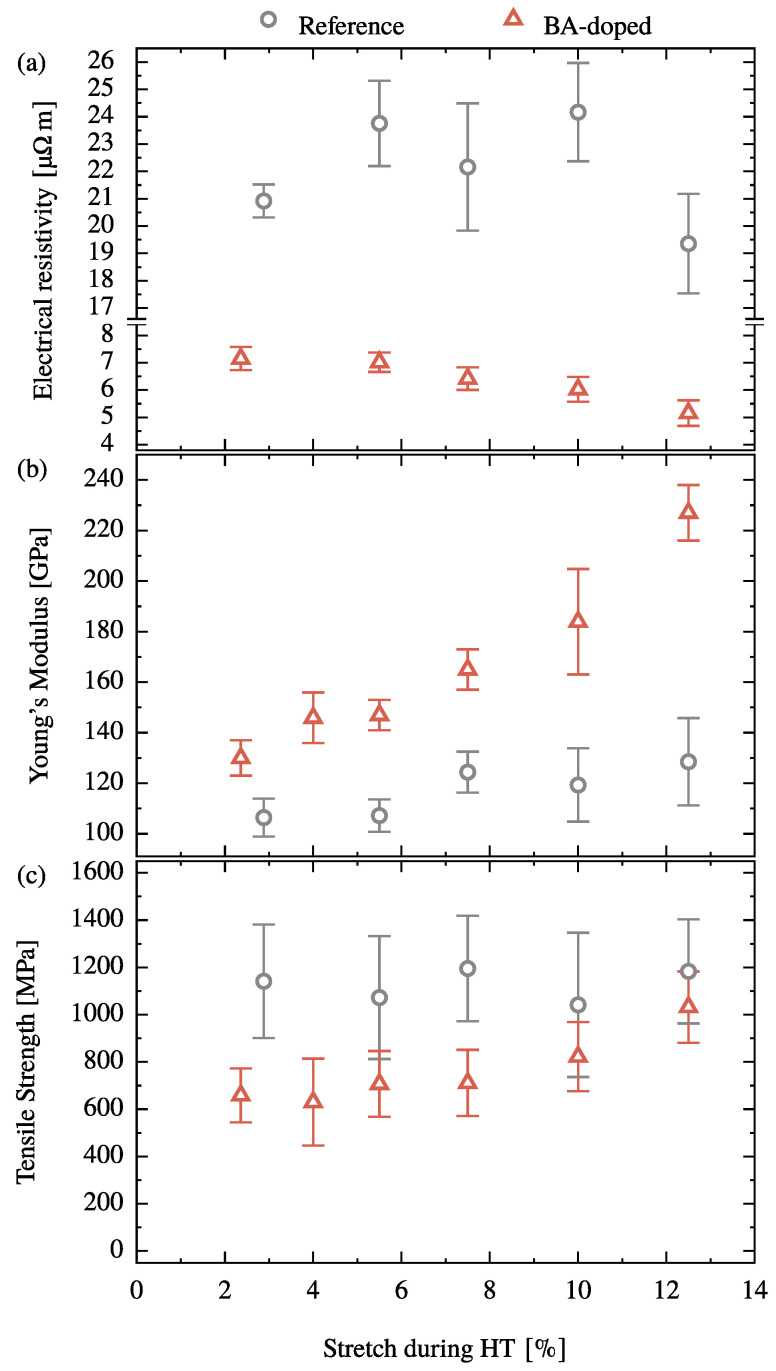
Electrical resistivity (**a**), Young’s modulus (**b**), and tensile strength (**c**) of the CFs made from the cellulose precursor (Reference) and the BA-doped precursor processed at different stretch ratios during carbonization at 2000 °C.

The electrical resistivity of the CFs is affected by both the stretch during the HT carbonization as well as by the BA-doping of the precursor itself (Figure 6a). The effect of the latter is more pronounced, as the resistivity is reduced by a factor of three as a consequence of the doubled lateral crystallite size for the BA-doped system [51]. The applied stretch systematically reduces the electrical resistivity of the BA-doped system even further. At a draw ratio of 12.5%, CFs with a resistivity of 5.1 μΩm were produced. This effect is not only caused by the additional stretch-induced crystallite growth, but also by the improved orientation, which increases the projection of the lateral crystallite size along the fiber axis. For the reference system, a similar trend was not observed. This can be explained on the one hand by the lower increase in preferred orientation and, on the other, by the smaller crystallites, whose alignment has a significantly less effect on resistivity.

The Young’s modulus scales almost linearly with the stretch during HT carbonization for both systems. This trend can be attributed to increased orientation, as reported and explained by different groups [9,32,52,53]. Since the increase in orientation is more pronounced for the BA-doped system than for the reference system, the same holds true for the Young’s modulus. In comparison with the samples processed tension-free, the Young’s modulus at 12.5% is increased by 22% up to 129 GPa and by 78% up to 230 GPa for the reference and the BA-doped systems, respectively.

The tensile strength stays constant at a level of 1200 MPa for the reference system and increases slightly from 700 MPa to 1000 MPa for the BA-doped system with applied stretch. Considering the higher orientation of the BA-doped system, the reduced tensile strength was not expected but can be explained by the presence of crystalline boron carbide particles. Evidence for these particles is provided by SEM–EDX analysis, where representative point spectra (Figure 2d) of surface agglomerates (Figure 2c) reveal a locally elevated boron concentration. Furthermore, WAXS analysis (Section 3.2.1) confirms the specific phase configuration as B_4_C. The additional phase can be considered a defect that initiates cracks and thus limits strength.

The elongation at break of both systems is largely unaffected by the applied stretch during carbonization, remaining at approximately 0.8% for the reference system and 0.5% for the BA-doped system.

### 3.4. Microstructural Analysis Using a Uniform Stress Model

To investigate the increased orientability of the CFs based on the BA-doped precursor, a uniform stress model was applied [52]. Northolt et al. demonstrated that a simplified version, a two-parameter approach (Equation (6)), adequately describes the relation between orientation and Young’s modulus of different CF types [54]. By using Equation (6), the microstructural modulus of the graphene layers in the direction normal to the c-axis ($e_{1}$) and the shear modulus normal to the c-axis (*g*) can be determined:

(6) $$\frac{1}{E_{\mathrm{corr}}}=\frac{1}{e_{1}}+\frac{\langle \cos^{2}\phi \rangle }{g}$$

Before Equation (6) was applied to the data of the two CF series, the Young’s moduli were corrected in terms of porosity by using Equation (7).

(7) $$E_{\mathrm{corr}}=\frac{\rho^{G}\;d_{002}^{G}}{\rho \;d_{002}}\;E$$

**Figure 7:**
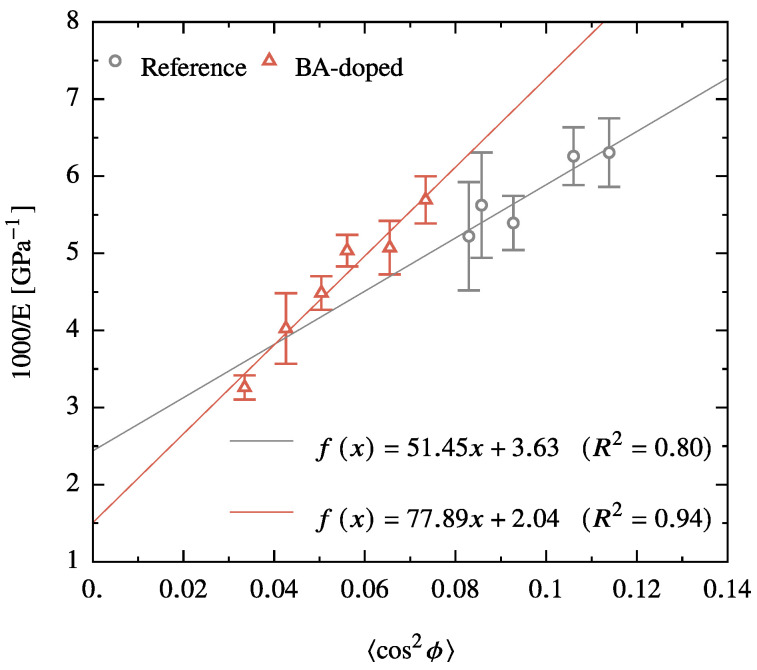
Correlation between the inverse Young’s modulus and the orientation of the CFs made from the cellulose precursor (Reference) and the BA-doped precursor processed at different stretch ratios during carbonization at 2000 °C.

Here, density and lattice plane spacing of single-crystal graphite ($\rho^{G}=2.265\;g/{cm}^{3}$ [55], $d_{002}^{G}=0.3354\;nm$ [41]) and the values for the present CFs were used (Table 2). The correction of the moduli yields scaling factors of 1.49 and 1.35 for the reference and BA-doped systems, respectively. According to Equation (6), the corrected inverse Young’s moduli are plotted as a function of the second moment of orientation (Figure 7).

Each set of pairs was fitted with a linear regression, and the corresponding values for $e_{1}$ and *g* are listed in Table 4.

For comparison, the values of Rayon-based CFs from Zhang et al. are given [32]. These samples are viscose-based CF, which were post-processed at different HT temperatures and analyzed in the same manner. For these samples $e_{1}$ is slightly increased and *g* is considerably lowered when the temperature during the HT carbonization is increased from 2100 to 2700 °C. In the literature, the following structural model is given, to explain the trend [30,32]: The shear modulus of the turbostratic carbon phase is proportional to the density of crosslinks, which are mostly present in cellulose-based CFs in form of sp^3^-hybridized C−C bonds. Due to their thermal instability, the number of crosslinks and therefore the shear modulus *g* can be reduced by increasing the temperature during HT carbonization. Since in single-crystal graphite no crosslinks are present, the smallest possible shear modulus was determined to be 5 GPa [56].

The moduli $e_{1}$ and *g* of the reference system differ remarkably from those for the Rayon-based CFs from Zhang et al. [32]. These deviations are most likely caused by the differences in precursors and CF processing. The samples from Zhang et al. are based on a viscose-based CF pre-heat-treated at 1300 °C and subsequently post-heat-treated by Zhang et al. [32].

Therefore, a comparison between the CFs based on the untreated and the BA-doped precursor seems to be more suitable. In doing so, significantly larger values for the in-plane modulus $e_{1}$ and smaller values for the shear modulus *g* are obtained when BA is used. BA doping affects $e_{1}$ and *g* in the same manner as does an increased carbonization temperature. If one assumes the reference system as the status quo, the changes within the context of the model can be interpreted as follows: BA is able to reduce the number of crosslinks and therefore lower the shear modulus. Consequently, the lattice plane spacing decreases, and the growth of crystallites is favored (Table 2), resulting in a higher electrical conductivity (Figure 6a). In such a system, the crystallites can align at lower tension during carbonization (Figure 3), giving CFs with higher Young’s moduli (Figure 6b).

A similar effect of boron-containing substances on the formation of ordered structures starting below 1900 °C was shown previously only for BA in polyethylene-derived CFs [30], even though different boron-containing substances and also BA itself have been investigated several times as an additive [57,58,59,60]. We assume that a strong interaction of BA with the precursor (cellulose or sulfonated polyethylene) sets these two systems apart from the other investigated systems.

## 4. Conclusions

A more energy-efficient process for making cellulose-based carbon fibers (CFs) involving boric acid (BA) treatment and stretch carbonization was demonstrated to achieve the properties of commercially available standard modulus CFs. In this way, the otherwise necessary energy-intensive UHT (ultra-high temperature) step can be avoided. By incorporating BA into the cellulose precursor, the orientability during carbonization is significantly enhanced, enabling effective carbonization below 2000 °C. To explain the role of BA, a uniform stress model was applied. If a BA-doped precursor is used instead of an untreated one, the resulting CFs produced at 2000 °C exhibit a reduction in shear modulus *g* from 29.0 to 17.3 GPa and an increase in the in-plane modulus $e_{1}$ from 410 to 664 GPa. This change is proposed to be caused by the reduction of cross-links between basal planes. As a direct result, the lattice plane spacings reduce to $d_{002}=0.3404\;nm$, and the crystallite growth is enhanced from $L_{a}=6\;nm$ to 11 nm. At the same time, the orientability during the stretch carbonization at 2000 °C is substantially increased. The optimized orientation of the crystallites reduces the electrical resistivity by over 30% down to 5.2 μΩm, while the Young’s modulus increases by 78% up to 230 GPa. For the undoped reference, the electrical resistivity (19.4 μΩm) and the Young’s modulus (125 GPa) change only slightly during the carbonization under tension. For these reasons, BA occupies a special place among other additives commonly used for cellulose-based CFs, as it increases the carbon yield [25] and enables a reduction in the carbonization temperature at the same time.

## Figures and Tables

**Table 1:** Characteristics of the cellulose (Reference) and the BA-doped precursor used for the manufacturing of CFs.

Sample-ID	Reference	BA-Doped
Spinning process	Viscose	Viscose
Number of filaments	1000	1000
Yarn linear density [tex]	294±5	311±5
Single filament linear density * [dtex]	2.9±0.1	3.1±0.1
Single filament cross-section * [μm^2^]	200±11	208±10
Single filament diameter ^1^ [μm]	16.0±3.7	16.3±3.6
Boron content * [wt%]	0.0±0.1	1.0±0.1
BA content ^2,^* [wt%]	0.0±0.1	5.6±0.1
OG ^3^	0.80	0.79

^1^ Calculated assuming round cross-sections, ^2^ calculated from boron content, ^3^ orientation degree (Equation (3)), * [25].

**Table 2:** Crystalline dimensions of the CFs made from the cellulose precursor (Reference) and the BA-doped precursor processed at different stretch ratios during HT carbonization at 2000 °C.

Reference					BA-Doped				
Stretch	Tension	$L_{a}$	$L_{c}$	$d_{002}$	Stretch	Tension	$L_{a}$	$L_{c}$	$d_{002}$
[%]	[cN/tex]	[nm]	[nm]	[nm]	[%]	[cN/tex]	[nm]	[nm]	[nm]
2.9 *	0.0	5.36	1.49	0.3507	2.4 *	0.0	9.58	4.85	0.3404
5.5	3.7	6.08	1.58	0.3512	5.5	2.9	10.19	5.15	0.3404
7.5	7.3	6.55	1.73	0.3513	7.5	4.8	11.11	5.44	0.3409
10.0	10.9	6.88	1.83	0.3513	10.0	6.3	10.22	5.31	0.3407
12.5	13.9	6.24	1.71	0.3513	12.5	9.8	11.19	5.54	0.3410

* Tension-free processed samples.

**Table 3:** Crystalline orientation of the CFs made from the cellulose precursor (Reference) and the BA-doped precursor processed at different stretch ratios during HT carbonization at 2000 °C.

Reference					BA-Doped				
Stretch	Tension	Γ	OG	$\langle \cos^{2}\phi \rangle$	Stretch	Tension	Γ	OG	$\langle \cos^{2}\phi \rangle$
[%]	[cN/tex]	[%]			[%]	[cN/tex]	[°]		
2.9 *	0.0	46.8	0.7502	0.1139	2.4 *	0.0	37.18	0.7962	0.0734
5.5	3.7	45.07	0.7510	0.1060	5.5	2.9	32.37	0.8181	0.0561
7.5	7.3	42.00	0.7686	0.0927	7.5	4.8	30.65	0.8310	0.0504
10.0	10.9	40.31	0.7791	0.0857	10.0	6.3	28.11	0.8429	0.0426
12.5	13.9	39.62	0.7852	0.0829	12.5	9.8	24.87	0.8625	0.0335

* Tension-free processed samples.

**Table 4:** Comparison of $e_{1}$ * and *g* ** determined by using a uniform stress model (Equation (6)) for the CFs based on the untreated (Reference), BA-doped precursor (BA), and values for Rayon-based CF, carbonized at different temperatures [32].

System	$e_{1}$ *	*g* **
	[GPa]	[GPa]
Reference ( 2000 °C)	410	29.0
BA-doped ( 2000 °C)	664	17.3
Rayon ( 2100 °C) [32]	650	22.8
Rayon ( 2300 °C) [32]	670	19.0
Rayon ( 2500 °C) [32]	680	16.9
Rayon ( 2700 °C) [32]	695	14.9

* $e_{1}$: Tensile modulus of the graphene layers in the direction normal to the c-axis. ** *g*: Shear modulus of the graphene layers normal to the c-axis.

## Data Availability

The data that support the findings of this study are available from the corresponding author, upon reasonable request.
